# Spatial Dosimetric-Based Prediction of Long-Term Urinary Toxicity After Permanent Prostate Brachytherapy

**DOI:** 10.3390/cancers18081287

**Published:** 2026-04-18

**Authors:** Chaoqiong Ma, Ying Hou, Rajeev Badkul, Jufri Setianegara, Xinglei Shen, Jay Shiao, Harold Li, Ronald C. Chen

**Affiliations:** 1 Department of Radiation Oncology, University of Kansas Medical Center, Kansas City, KS 66160, USA; 2 Department of Radiation Oncology, University of Pennsylvania, Philadelphia, PA 19104, USA

**Keywords:** prostate LDR, urinary toxicity prediction, spatial dose distribution, IPSS resolution

## Abstract

Background: To explore the correlation between spatial dose distribution and post-implant urinary toxicity, aiming to assist decision making in low-dose-rate (LDR) treatment planning, thereby improving patient outcomes. Methods: Eighty-five prostate LDR patients with >12-month follow-up were included. Patient-reported urinary toxicity was collected prospectively using the International Prostate Symptom Score (IPSS) questionnaire, from before implant (baseline) to post-implant follow-up. Patients were then grouped into those whose symptom scores returned to ≤2 points above baseline by 12 months (no long-term toxicity) vs. those who did not (long-term toxicity). A total of 106 features were extracted for each patient, including principal components of dose-volume histograms (DVHs) from multiple prostate subzones, the whole prostate and urethra, along with baseline IPSS, implantation characteristics, and additional DVH indicators for the prostate and the urethra. A machine learning (ML) model incorporating backward feature selection algorithm was developed to predict long-term toxicity status, using a shuffle-and-split validation strategy for model evaluation during feature selection. A univariate statistical analysis was conducted on the model’s selected features. Results: Out of 85 patients, 41 (48%) had long-term urinary toxicity. Seven features were selected during model training, including baseline IPSS and six dosimetric features from several prostate subzones primarily located in the posterior prostate. The model achieved a high mean area under the receiver operating characteristic curve (AUC) of 0.81, with a balanced sensitivity and specificity of 0.78 by adjusting the probability threshold. In univariate analysis, only baseline IPSS and one selected dose feature were significantly correlated with long-term toxicity with AUC < 0.71. Conclusions: The proposed ML model, integrating baseline IPSS and spatial dosimetric features, effectively predicts long-term urinary toxicity after prostate LDR. This approach offers a practical method for risk stratification, allowing clinicians to identify patients at elevated risk and prioritize them for targeted preventative measures and closer follow-up.

## Simple Summary

Despite excellent local control and disease-free survival achieved with low-dose-rate (LDR) brachytherapy for prostate cancer, a wide range of acute and late toxicities remain a concern. Early identification of patients at higher risk of post-implant urinary toxicity can facilitate targeted preventive strategies and closer follow-up. In this study, we characterized the spatial dose distribution from LDR seed implantation in patients receiving combined LDR and external beam radiotherapy (LDR-EBRT) and integrated these features with clinical and implantation-related parameters to develop a machine learning model for urinary toxicity prediction. The proposed model, incorporating baseline International Prostate Symptom Score and spatial dosimetric features, demonstrated strong predictive performance, with an area under the receiver operating characteristic curve of 0.81. This model provides a practical approach for post-implant risk stratification in prostate cancer patients undergoing LDR-EBRT.

## 1. Introduction

Permanent low-dose-rate (LDR) seed brachytherapy is a well-established and effective treatment for localized prostate cancer, offering targeted dose escalation to the prostate with rapid dose fall-off to spare surrounding normal tissues [1]. For low- and intermediate-risk patients, LDR monotherapy provides excellent long-term biochemical control [2]. In unfavorable intermediate- and high-risk cases, international guidelines recommend combining LDR with external beam radiotherapy (LDR-EBRT) to deliver a high total dose while reducing the required EBRT dose [3,4].

Despite the excellent local control and disease-free survival rates reported for both LDR monotherapy and LDR-EBRT, a wide range of acute and late toxicities have been observed with LDR-involved treatments [5,6,7]. Patient-reported outcomes indicate that LDR-EBRT is associated with significantly worse late genitourinary (GU) toxicity compared to EBRT alone, including declines in overall function, increased irritative symptoms, and urinary frequency [8,9,10,11,12]. Additionally, clinician-assessed outcomes have shown that LDR-EBRT results in significantly higher rates of late grade ≥3 (G3+) GU toxicity than EBRT alone [13,14,15].

Several predictive factors such as baseline International Prostate Symptom Score (IPSS), prostate volumes, association with neoadjuvant androgen deprivation therapy (ADT), and seed density have been reported to be associated with urinary toxicities in LDR treatment [5,6,7,16]. Additionally, the dose to the most exposed 2 cm^3^ (D_2cc_) of the bladder neck showed strong prognostic value for both acute and late urinary toxicity [17]. For LDR-EBRT specifically, age, Gleason score, and various IPSS thresholds were each predictive of G2+ urinary toxicity, while dose-volume-histogram (DVH) metrics measuring prostate dose coverage for planning were not [18]. In contrast, for LDR monotherapy, significant predictors included higher baseline IPSS, maximal post-implant IPSS, presence of acute toxicity, and a larger prostate volume receiving ≥ 150% prescription dose (V_150%_) [19]. Furthermore, spatial dose distribution within the prostate, divided into four quadrants, was evaluated for its association with disease relapse; however, neither regional nor whole-prostate DVH metrics showed a statistically significant correlation with relapse [20,21].

In recent years, machine learning (ML) approaches have been increasingly applied in radiation oncology for outcome prediction, enabling the integration of diverse data sources such as clinical characteristics, treatment parameters, imaging and dosimetric features [22]. These methods are particularly well suited for modeling complex and potentially non-linear relationships among variables, offering improved predictive performance compared with traditional statistical approaches [23,24]. ML-based outcome prediction models have shown promise in identifying patients at elevated risk of treatment-related toxicity, thereby supporting personalized treatment strategies and risk-adapted patient management.

To our knowledge, there is a lack of studies examining the combined influence of spatial dose distribution and clinical factors on GU toxicity in LDR-EBRT brachytherapy. In this study, we aimed to assess the correlation between long-term urinary toxicity following LDR-EBRT and spatial dose characteristics, relevant DVH metrics, clinical and implantation-related parameters. We characterized the spatial dose distribution from LDR seed implantation and combined it with other features to develop an ML-based model for urinary toxicity prediction. This model is intended to support clinical decision-making by identifying patients at higher risk of post-implant urinary toxicity, enabling targeted preventive strategies and closer follow-up. This work provides a comprehensive framework that integrates spatial dosimetric and clinical factors for toxicity prediction and offers a practical approach for post-implant risk stratification in LDR-EBRT.

## 2. Methods and Materials

### 2.1. Treatment Characteristics and Patient Cohort

At our institution, LDR prostate brachytherapy is performed using loose I-125 seeds (Best Medical model # 2301; Best Medical International, Springfield, VA, USA), implanted via a real-time, transrectal ultrasound (TRUS)-guided transperineal technique. Implantation locations and treatment planning are determined intraoperatively using the VariSeed treatment planning system v09.00.30 (Varian Medical Systems, Palo Alto, CA, USA), based on real-time contouring of the clinical target volume (CTV, defined as the prostate) and organs at risk, including the urethra and rectum. Dose calculations were performed using the TG-43 formalism in the VariSeed treatment planning system. With the patient under general anesthesia in the lithotomy position, TRUS guidance is facilitated by a CIVCO EX3 electronic stepper and CIVCO Micro-Touch stabilizer unit (CIVCO Medical Solutions, Coralville, IA, USA). A Mick applicator (Mick Radio-Nuclear Instruments, Mt Vernon, NY, USA) is used to implant the peripheral seeds first, followed by interior seed placement through applicator needles.

This retrospective study was conducted with approval from the Institutional Review Board. We included 85 consecutive patients (median age: 70 years) treated at our institution between January 2021 and December 2023 for unfavorable intermediate- or high-risk prostate cancer. All patients received LDR brachytherapy prescribed to 110 Gy plus external beam radiotherapy (EBRT) to 45 Gy, typically starting six weeks post-implantation. The minimum follow-up period was 12 months. Patient characteristics and treatment parameters are summarized in Table 1.

The intraoperative planning goal was to achieve D_90%_ ≥ 100%, ensuring that at least 90% of the prostate volume received the 100% prescription dose. The volume receiving over 150% of the prescription dose (V_150%_) was constrained to less than 50% to minimize hot spots. For organs at risk, the rectal wall V_100%_ was limited to 1 cc, and the maximum dose to the urethra was restricted to 150% of the prescription dose.

### 2.2. Outcome Measurement

The IPSS questionnaire was prospectively collected for all patients at baseline (prior to brachytherapy) and at scheduled follow-up visits to assess urinary symptom severity, with higher scores indicating greater symptom burden. Post-treatment follow-up were typically performed every 3–6 months. All patients included in this study had at least 12 months of IPSS follow-up data.

It is common for patients to experience a transient increase in IPSS following implantation, reflecting worsened urinary symptoms, followed by gradual improvement over time. Prior studies have shown that approximately 85% of patients undergoing LDR brachytherapy achieve IPSS resolution—defined as a return of the post-implant IPSS to within 2 points of baseline, indicating a return to near baseline urinary function while accounting for normal score variability [25]—with a median time to resolution of approximately 12.6 months [19,26]. Accordingly, 12-month IPSS resolution was used as the endpoint for long-term urinary toxicity. Patients who achieved IPSS resolution within 12 months were classified as having no long-term toxicity, while those who did not were classified as experiencing long-term urinary toxicity, with symptoms persisting beyond 12 months or potentially permanent.

### 2.3. Spatial Dose Characteristics

**Figure 1:**
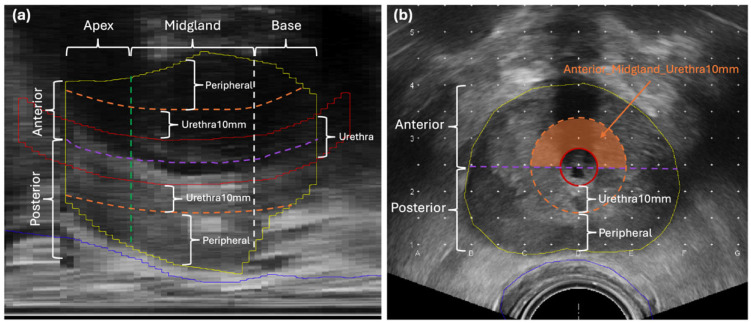
Prostate segmentation in mid-sagittal (**a**) and mid-axial (**b**) views of an ultrasound image for spatial dose characterization. The prostate, urethra and rectum are contoured in yellow, red and blue solid lines, respectively. The purple dashed line represents the coronal bisector of urethra, defining the anterior zone (anterior to the line) and posterior zone (posterior to the line). The annular region between the orange dashed contour and the urethral contour defines the urethra-near zone (Urethra10mm). The green and white dashed lines in (**a**) divide the prostate longitudinally into the apex (inferior to the green line), mid-gland (between the green and white lines), and base (superior to the white line). The orange-shaded region in (**b**) illustrates an example of a composite subzone, located in the urethra-near region of the anterior mid-gland.

To characterize the spatial dose distribution of the implantation plan, the prostate and urethra were segmented into multiple subzones, with dose statistics evaluated across both individual subzones and their composite subzones. The segmentation approach was adopted from a previous study on seed dynamics within the prostate [27,28], in which the prostate was divided along the superior–inferior axis into base, midgland, and apex using a 1:2:1 ratio, and further subdivided into anterior and posterior regions based on the urethral midpoint, as well as into central and peripheral regions using a 1 cm radius circle centered on the urethra. This scheme was originally developed to evaluate I-125 seed dynamics and demonstrated region-dependent variations, supporting its clinical relevance. The resulting subzones are relevant to treatment planning decisions aimed at achieving adequate CTV dose coverage while sparing the urethra and rectum, as illustrated in Figure 1a,b.

In our implementation, we adopted and refined this scheme to better capture dose heterogeneity around the urethra. Specifically, the prostate was divided into apex, midgland, and base zones along the superior-inferior axis in a 1:2:1 ratio. Further subdivisions were made based on the position of the urethra. The anterior and posterior zones were defined in each axial slice relative to the urethral midpoint: the region anterior to the urethral center was designated as the anterior zone, while the remaining prostate tissue was classified as the posterior zone. The urethra was approximated as a circle with a 7 mm radius in each axial slice. In addition, a urethra-near region (Urethra10mm) was defined as the annular volume between 7 mm and 10 mm radial distance from the urethral center, and the peripheral zone was defined as the remaining prostate volume beyond this region. The urethra itself was treated as a separate subzone.

Subsequently, the spatial dose statistics for each subzone were characterized using dose–volume histograms (DVHs) derived from the implantation plan—for example, the DVHs of the anterior and peripheral zones. In addition, DVHs were extracted for composite subzones formed by the intersection of two or three subzones, such as the intersection of the anterior, mid-gland, and Urethra10mm zones, illustrated as the orange-shaded area in Figure 1b. A complete list of the 48 individual and composite subzones is provided in Table 2. The DVHs from these relatively smaller composite subregions enabled capturing more localized dose features, facilitating more precise identification of regions potentially associated with urinary toxicity. And the inclusion of both individual and composite subzone DVHs was intended to capture potential combinatorial effects relevant to urinary toxicity prediction in the subsequent multivariate modeling.

To reduce feature dimensionality while preserving the most significant variation patterns in the dose distribution, Principal Component Analysis (PCA) was applied to each DVH. The first two principal components, denoted as [zone name]_PCA1_ and [zone name]_PCA2_, were obtained for each DVH, explaining about 95% of the variance data [29,30]. In total, 96 PCA-derived features were obtained for each patient, characterizing the spatial dose distribution of the implantation plan.

### 2.4. Additional Dosimetric, Implantation-Related and Clinical Parameters

In addition to the spatial dose distribution characteristics, ten additional relevant dosimetric, implantation-related and clinical indicators were extracted for each patient, as summarized in Table 3. Specifically, the dosimetric parameters included several DVH indicators for the whole prostate and the urethra: prostate D_90%_, V_100%_, and V_150%_, and urethra D_5%_, D_30%_, and V_150%_. The implantation-related parameters potentially associated with post-implant urinary toxicity, including the total number of needles (as a surrogate for prostate trauma), total number of seeds, and prostate volume, were also collected. Furthermore, one clinical indicator, baseline IPSS, was incorporated.

### 2.5. ML-Based Prediction Model

To identify significant predictors of post-implant urinary toxicity, we established a multivariate prediction model using a machine learning technique to correlate the spatial dose characteristics, as well as relevant dosimetric, implantation-related and clinical indicators, with long-term urinary toxicity. The model employed a support vector machine classifier with a radial basis function kernel (SVM-RBF), which projects input features into a higher-dimensional space to account for potential nonlinear relationships. To enhance model generalizability and minimize the influence of redundant or non-predictive features, we incorporated a sequential backward selection (SBS) procedure that iteratively pruned the feature set, maximizing the area under the receiver operating characteristic (ROC) curve (AUC).

**Figure 2:**
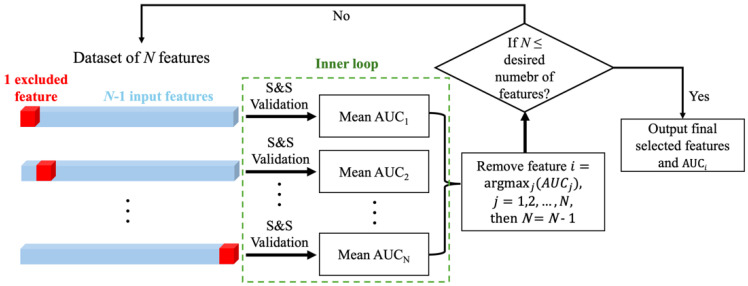
The nested validation architecture of the modeling framework. The outer loop implements SBS for feature elimination, while the inner loop (green dashed line) employs S&S validation strategy to assess feature importance. At each iteration of SBS, the least important feature *i* is identified through the inner loop of S&S validation strategy and removed from the current set of *N* features. The process terminates when reaching the desired number of features, outputting both the final feature subset and corresponding model performance AUC*_i_*.

As shown in Figure 2, the modeling framework implemented a nested validation architecture: the outer loop implemented the SBS procedure, and the inner loop employed a shuffle-and-split (S&S) validation strategy during each SBS iteration to assess feature importance. During each SBS iteration, the model (SVM-RBF) was trained, and the performance (quantified by AUC) was evaluated across multiple random splits of the complete dataset for every candidate feature subset (obtained by excluding one feature at a time from the current feature set). The feature whose exclusion resulted in the highest AUC value was identified as the least important and subsequently removed. To minimize bias from data partitioning, the whole dataset was shuffled and split 1000 times independently with the mean AUC across all splits serving as the robust criterion for feature elimination decisions. A total of 1000 iterations was chosen to ensure stable performance estimates and reduce variability associated with random partitioning, while maintaining computational efficiency. The validation/SBS process was terminated once the number of remaining features reached a desired level, in accordance with the one-in-ten rule, ref. [31], ensuring it did not exceed one-tenth of the dataset size. For instance, in a cohort of 100 patients, no more than 10 features would be retained while maximizing model performance. Further details of this framework can be found in the study by Ma et al. [32].

The SVM-RBF model was implemented using the publicly available Python package scikit-learn v1.3.2 [33]. Hyperparameter optimization (HPO) was performed using the particle swarm optimization (PSO) algorithm [34], as implemented in Optunity [35], to identify the optimal regularization parameter C (C ∈ [0, 100]) and the kernel coefficient γ (γ ∈ [10^−5^, 100]).

It is worth noting that prior to the SBS procedure, all features were scaled using min-max normalization, after which a Pearson correlation test was performed to identify and eliminate highly collinear features (r > 0.9). And the remaining multicollinearity of the features was further addressed by SBS procedure to ensure model stability by mitigating the risk of overfitting associated with feature redundancy.

### 2.6. Univariate Statistics Analysis

Complementing our multivariate analysis, we performed univariate analysis (UVA) using Spearman’s rank correlation to evaluate all 106 extracted features for individual associations with long-term urinary toxicity. Features demonstrating significant correlations (*p* < 0.05) were identified, and their individual performance in predicting long-term urinary toxicity was evaluated. This analysis served two main purposes: (1) to identify features demonstrating sufficient predictive performance (AUC > 0.65) for potential clinical implementation as standalone markers, and (2) to distinguish between features exhibiting independent predictive value versus those requiring multivariate interactions for significance.

## 3. Results

### 3.1. Model Performance and Selected Features

**Figure 3:**
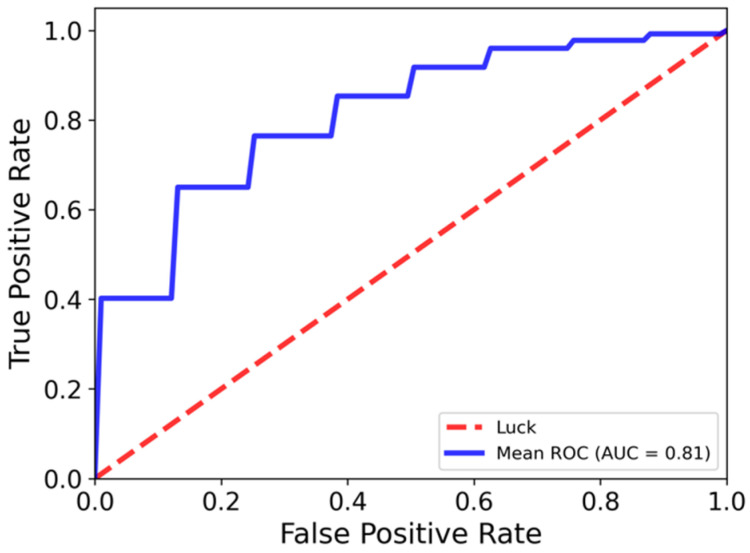
ROC curve illustrating the model’s predictive performance for long-term urinary toxicity. The mean ROC curve (blue) achieved an AUC of 0.81. The red dashed line represents the reference line for random classification.

Out of the 85 patients included in the study, 41 (48%) experienced long-term urinary toxicity. The performance of the prediction model in identifying patients at risk of toxicity is illustrated by the mean ROC curve across 1000 times of S&S validations in Figure 3. The model achieved robust predictive performance, yielding a mean AUC of 0.81 and an accuracy of 0.75. Additionally, balanced sensitivity and specificity were observed, measured at 0.77 and 0.73, respectively.

The modeling process was terminated when the eight most discriminative features were selected from the nested model training and feature elimination process. These features are listed in Table 4 in descending order of their importance in predicting long-term urinary toxicity. Feature importance was assessed by measuring the mean AUC across 1000 times of S&S validations after the removal of each feature from the selected feature set. A greater decrease in the mean AUC following feature removal (i.e., a lower resulting mean AUC) indicated higher importance of that feature for prediction.

**Figure 4:**
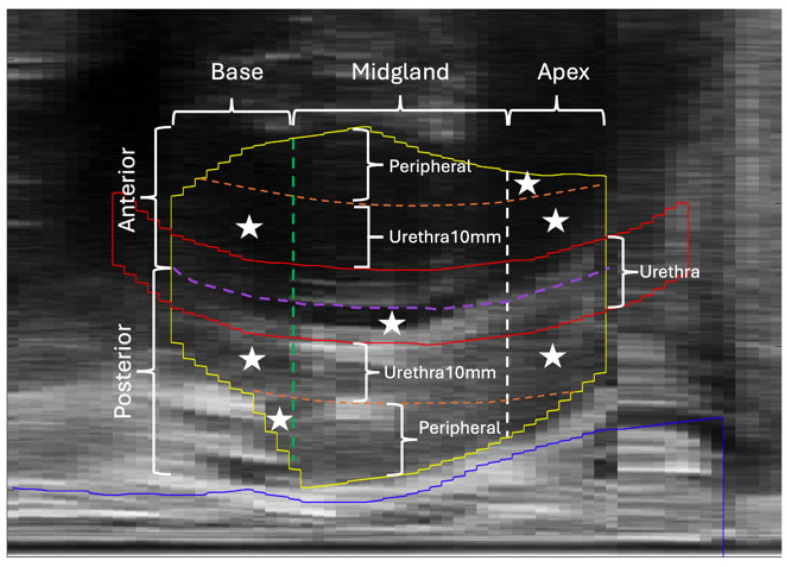
Prostate and urethra segmentations in mid-sagittal view of an ultrasound image, with white stars indicating seven composite subzones (listed in Table 4) contributing the most discriminative spatial dose features. The prostate, the urethra and the rectum are contoured in yellow, red and blue solid lines, respectively. The purple dashed line represents the coronal bisector of urethra, defining the anterior zone (anterior to the line) and posterior zone (posterior to the line). The annular region between the orange dashed contour and the urethral contour defines the urethra-near zone (Urethra10mm). The green and white dashed lines divide the prostate longitudinally into the apex (inferior to the green line), mid-gland (between the green and white lines), and base (superior to the white line).

Among all the selected features, only one was a clinical indicator—baseline IPSS—which emerged as the third most important predictor; its removal resulted in a significant reduction of 8.8% in the mean AUC. This finding is consistent with previous literature identifying baseline IPSS as a significant factor in predicting LDR urinary toxicity. The remaining seven features were spatial dose features derived from specific composite subzones, visualized in the mid-sagittal view of the segmented prostate and urethra in Figure 4. Overall, most of these features (five out of seven) were in or near the urethra, while only two originated from the peripheral prostate. Among the five features from the urethra or urethra-near zones (Urethra10mm), one was situated within the urethra of the posterior mid-gland and was the only feature derived from the mid-gland. The remaining four were distributed across the urethra-near zones in the base and apex. Notably, a dose feature from the urethra near zone of apex (Apex_Urethra10mm_PCA1) was identified as the most important feature, with its removal leading to a substantial reduction of 11.7% in the mean AUC. In contrast, the two dose features from the peripheral subzones of the base and apex had relatively less impact on predictive performance, with the removal of either feature resulting in a mean AUC reduction of around 4–7%. In addition, none of the extracted DVH indicators for the whole prostate and urethra, nor the implantation-related parameters, including the total numbers of needles and seeds, and prostate volume, were selected as discriminative features.

Notably, none of the conventional whole-organ DVH metrics for the prostate or urethra were selected as predictive features. This may be because these metrics summarize dose at the whole organ level and do not capture the spatial heterogeneity of dose distribution. In addition, current clinical planning enforces relatively consistent target coverage and organ-at-risk constraints, reducing inter-patient variability in conventional DVH parameters and thereby limiting their discriminative ability.

### 3.2. Results of Univariate Statistics Analysis

Out of the 106 extracted features, three were found to be statistically significantly associated with long-term urinary toxicity in LDR patients based on UVA. Table 5 presents these significant features alongside an additional three features that demonstrated notable trends toward significance (*p* < 0.1). Among the three significant features, only one feature—baseline IPSS—was also selected as a discriminative predictor by the multivariate machine learning-based model. Specifically, baseline IPSS exhibited the strongest association with the outcome, reflected by the smallest *p*-value and the highest predictive performance among the three features, with an AUC of 0.708. In addition, baseline IPSS was negatively correlated with IPSS resolution status, suggesting that patients with higher baseline scores were less likely to develop long-term urinary toxicity. This finding aligns with prior studies reporting that baseline urinary symptoms may improve following radiation therapy. Although an additional two features demonstrated statistically significant associations with the outcome and three others approached significance, their individual predictive performance was limited (AUC < 0.63), suggesting insufficient value as standalone clinical markers.

The multivariate ML model outperformed univariate analysis (AUC 0.81 vs. 0.708). Notably, seven of the eight selected features were not significant in univariate analysis, indicating that their predictive value arises from interactions among variables. These results highlight the ability of multivariate modeling to capture complex relationships for robust prediction. Accordingly, the proposed model, integrating baseline IPSS with spatial dosimetric features, shows strong potential for predicting long-term urinary toxicity following prostate LDR brachytherapy.

## 4. Discussions

In the present study, baseline IPSS along with seven spatial dose features of the prostate and urethra demonstrated strong predictive performance for urinary toxicity in patients who underwent LDR brachytherapy plus supplemental EBRT. Similarly, Eriguchi et al. [7], using multivariate analysis, reported that faster IPSS resolution was associated with higher baseline IPSS (consistent with our findings), fewer needles, and lower urethral D_30%_, while grade ≥2 urinary toxicity correlated with baseline IPSS, ADT, and seed density. Unlike our approach, their analysis included only selected DVH parameters of the whole prostate and urethra—typically used as planning constraints—which provide limited characterization of spatial dose distribution. Hamilton et al. [20] examined the correlation of regional quadrant dose with late GU toxicity, finding that higher doses to the anterior prostate were associated with increased grade ≥2 GU toxicity. However, even the best-performing regional DVH predictors yielded modest ROC AUC values. In contrast, our study employed finer segmentations of both the prostate and urethra to characterize spatial dose distribution more comprehensively, resulting in substantially improved predictive performance of the ML-based model for late urinary toxicity. It is important to note that direct comparisons with these studies are limited as they assessed urinary toxicity differently and included patients treated with LDR monotherapy without EBRT.

It is noteworthy that all predictive spatial dose features in the proposed model were derived from six unique composite subzones—primarily located in or near the urethra within the anterior and posterior prostate—rather than from the initial subzones (e.g., apex prostate or urethra10mm) or the entire prostate and urethra. These composite subzones represented relatively small, intersecting regions formed by overlapping initial subzones. Among these selected composite subzones, two exhibited an inclusion relationship: the urethra near-zone in the anterior apex (Apex_Anterior_Urethra10mm) was a subset of the anterior apex urethra zone (Apex_Urethra10mm). This suggests that highly localized spatial dose features can carry strong predictive value for urinary toxicity. In particular, features from the composite subzones with inclusion relationships may reflect overlapping anatomical sensitivities, amplifying their predictive contribution. Their combined influence may capture complex spatial dose–response patterns that are not apparent from single regions alone. It is interesting to note that the predictive significance of these dose features from the urethra near-zone in the apex in our model could possibly reflect the dose-sensitivity of the external urethral sphincter (EUS), located immediately distal to the prostatic apex. Previous studies have demonstrated that several dosimetric variables of EUS are correlated with increased toxicity and urethral stricture [36,37]. As the importance of this region is well-recognized in clinical practice, the American Brachytherapy Society consensus guidelines recommend that the EUS be explicitly contoured to ensure adequate sparing during treatment planning [38].

In this study, PCA was applied to transform the DVHs of each subzone into uncorrelated principal components. Although these components represent linear combinations of the original DVH metrics, they are not easily interpretable for direct treatment planning. This difficulty arises because the established ML-model is inherently non-linear, predicting clinical outcomes through a complex, non-linear combination of the selected dose features and baseline IPSS. On the other hand, the primary objective of this model was to maximize predictive accuracy for late urinary toxicity. To this end, we evaluated SVM-based ML models with linear and non-linear kernels (RBF and polynomial) and compared the performance of raw DVH metrics against PCA-derived DVH features, finding that the SVM-RBF model utilizing PCA-derived DVH features yielded the highest AUC. While identifying specific dose indicators to guide treatment planning would be ideal, our clinical protocol prioritizes sufficient dose coverage for local control while respecting established dose limits for the urethra, rectum, and bladder. For instance, patients with dominant intraprostatic lesions (DILs) in the apex region inevitably receive higher doses in that area compared to those with DILs located elsewhere. Given that current protocol achieves excellent local control rates of >90%, it remains unclear whether dose reduction in these predictive regions would compromise tumor control solely to improve toxicity outcomes. Thus, the primary clinical utility of this method lies in risk stratification rather than guiding treatment planning, providing a means to identify patients at high risk for late urinary toxicity based on their specific implant dosimetry.

Our ML-based model for predicting late urinary toxicity significantly outperformed the best-performing univariate model, highlighting the importance of capturing spatial dose interdependencies and incorporating clinical indicators to fully characterize the interplay between dose distribution and patient-specific factors in toxicity prediction. It is relevant to note that ML-based modeling generally offers greater flexibility and predictive capability than conventional multivariate analysis, which relies on predefined assumptions and typically models linear relationships with limited features. In contrast, our ML-based models capture complex, nonlinear interactions among high-dimensional dose and clinical variables without such assumptions. While conventional multivariate analysis validation depends on *p*-values and confidence intervals, our ML approach emphasizes predictive accuracy via cross-validation, offering a more robust assessment of generalizability.

The limitations of this study should be acknowledged. It is a single-institution study with a limited patient cohort, which may affect the generalizability of the prediction model. Although we applied multiple strategies to mitigate overfitting, including SBS procedure for feature reduction and an S&S validation strategy for model fitting and validation, external validation in larger, multi-institutional cohorts is necessary to confirm the robustness and generalizability of the model. Additionally, the limited follow-up duration constrained the assessment of long-term recovery in patients with late urinary toxicity, potentially underestimating symptom resolution over time. Future studies can apply this ML-based approach to larger multi-institutional datasets with extended follow-up to enable more robust validation and capture long-term toxicity and recovery patterns. Additionally, the proposed prediction model was developed specifically for patients treated with LDR brachytherapy plus EBRT, and not brachytherapy monotherapy. Our proposed method could be implemented in future studies to characterize spatial dose distribution and integrate clinical data for predicting urinary toxicity in LDR monotherapy patients.

The proposed prediction model could be integrated into the prostate LDR workflow to identify patients at elevated risk of post-implant urinary toxicity, enabling targeted prevention and closer follow-up. By incorporating spatial dose features and patient-specific clinical factors, it offers individualized risk stratification beyond conventional planning metrics. This, in turn, can inform clinical decisions such as initiating prophylactic interventions or prioritizing early symptom monitoring for patients at high risk of post-implant urinary toxicity. In clinical practice, this risk stratification could also support post-treatment patient counseling, guide follow-up intensity (e.g., more frequent assessments for high-risk patients), and facilitate earlier initiation of symptom-directed medical management, potentially accelerating recovery and reducing the likelihood of persistent toxicity. Furthermore, there is potential to enhance predictive accuracy by integrating post-implant dosimetry. While the current model relies on planning dosimetry, future investigations could extract features from post-implant dose distributions to capture the actual delivered dose. Training an ML model on these post-implant features, either independently or combined with planning data, may provide a more robust assessment of toxicity risk.

## 5. Conclusions

The proposed ML model, integrating baseline IPSS and spatial dosimetric features, effectively predicts long-term urinary toxicity after prostate LDR. By employing fine prostate segmentation to comprehensively characterize spatial dose distribution, the model achieves strong predictive performance. This approach offers a practical method for post-implant risk stratification, rather than informing treatment planning, allowing clinicians to identify patients at elevated risk and prioritize them for targeted preventative measures and closer follow-up.

Future work should focus on validating the robustness and generalizability of the model in larger, multi-institutional cohorts. Additionally, incorporating post-implant dosimetry to better reflect the delivered dose, as well as integrating multimodal data such as imaging features, may further improve predictive accuracy and support clinical translation.

## Figures and Tables

**Table 1:** Patient characteristics and treatment protocol.

	Parameter	Value/Description
Patient Demographics	Total Patients	85 (consecutive, January 2021–December 2023)
	Median Age	70 years
	Risk Group	Unfavorable Intermediate or High-Risk
Treatment Regimen	Modality	LDR Brachytherapy + EBRT
	LDR Prescription Dose	110 Gy
	EBRT Dose	45 Gy (start ~6 weeks post-implant)
	Follow-up	Minimum 12 months
Planning Goals	Prostate D90%	≥100%
	Prostate V150%	<50%
OAR Constraints	Rectal Wall V100%	≤1 cc
	Urethra Dmax	<150%

**Table 2:** List of 48 individual and composite subzones of the prostate.

Zone Category	Zone Names
Individual subzones	Prostate, Anterior, Posterior, Urethra, Urethra10mm, Peripheral, Base, MidGland, Apex
Base Region	Base_Anterior, Base_Anterior_Peripheral, Base_Anterior_Urethra, Base_Anterior_Urethra10mm, Base_Posterior, Base_Posterior_Peripheral, Base_Posterior_Urethra, Base_Posterior_Urethra10mm, Base_Peripheral, Base_Urethra, Base_Urethra10mm
Midgland Region	MidGland_Anterior, MidGland_Anterior_Peripheral, MidGland_Anterior_Urethra, MidGland_Anterior_Urethra10mm, MidGland_Posterior, MidGland_Posterior_Peripheral, MidGland_Posterior_Urethra, MidGland_Posterior_Urethra10mm, MidGland_Peripheral, MidGland_Urethra, MidGland_Urethra10mm
Apex Region	Apex_Anterior, Apex_Anterior_Peripheral, Apex_Anterior_Urethra, Apex_Anterior_Urethra10mm, Apex_Posterior, Apex_Posterior_Peripheral, Apex_Posterior_Urethra, Apex_Posterior_Urethra10mm, Apex_Peripheral, Apex_Urethra, Apex_Urethra10mm
Anterior Region	Anterior_Peripheral, Anterior_Urethra, Anterior_Urethra10mm
Posterior Region	Posterior_Peripheral, Posterior_Urethra, Posterior_Urethra10mm

**Table 3:** List of additional DVH, implant-based and clinical indicators.

Category	Parameters
DVH	Prostate (D_90%_, V_100%_, and V_150%_) Urethra (D_5%_, D_30%_, and V_150%_)
Implant-based	Number of needles Number of seeds Prostate volume
Clinical	Baseline IPSS

**Table 4:** The eight selected features listed in descending order of importance. The mean AUC for each feature reflects the model performance after its removal from the selected feature set. Feature names denote composite subzones formed by combining anatomical partitions (base/mid-gland/apex, anterior/posterior, and urethra/urethra-near [Urethre10mm]/peripheral zones).

Selected Features	Mean AUC
Apex_Urethra10mm_PCA1	0.693
Base_Urethra10mm_PCA2	0.714
Baseline IPSS	0.722
Apex_Anterior_Urethra10mm_PCA1	0.724
Apex_Anterior_Peripheral_PCA2	0.741
Base_Posterior_Peripheral_PCA2	0.765
MidGland_Posterior_Urethra_PCA2	0.767
Base_Urethra10mm_PCA1	0.781

**Table 5:** Univariate analysis of features associated with long-term urinary toxicity. Features showing statistical significance (*p* < 0.05) or a trend toward significance (*p* < 0.1) are listed along with their corresponding AUC values. Feature names denote composite subzones formed by combining anatomical partitions (base/mid-gland/apex, anterior/posterior, and urethra/urethra-near [Urethre10mm]/peripheral zones).

Significant Features	*p*-Value	AUC
Baseline IPSS	**<0.001**	0.708
Apex_Posterior_Urethra10mm_PCA1	**0.048**	0.625
Apex_Posterior_PCA1	**0.047**	0.624
Apex_Posterior_Peripheral_PCA2	0.054	0.621
Posterior_PCA1	0.067	0.615
MidGland_Posterior_Urethra_PCA1	0.070	0.625

## Data Availability

The data that support the findings of this study are not publicly available due to patient privacy and institutional ethical restrictions.
